# Systematic review with network meta-analysis: Comparative efficacy of oral nucleos(t)ide analogues for the prevention of chemotherapy-induced hepatitis B virus reactivation

**DOI:** 10.18632/oncotarget.8907

**Published:** 2016-04-21

**Authors:** Min-Yue Zhang, Gui-Qi Zhu, Ke-Qing Shi, Ji-Na Zheng, Zhang Cheng, Zhuo-Lin Zou, Hong-Hui Huang, Fang-Yuan Chen, Ming-Hua Zheng

**Affiliations:** ^1^ Department of Hematology, Renji Hospital, School of Medicine, Shanghai Jiaotong University, Shanghai 200127, China; ^2^ Department of Hepatology, Liver Research Center, the First Affiliated Hospital of Wenzhou Medical University, Wenzhou 325000, China; ^3^ School of the First Clinical Medical Sciences, Wenzhou Medical University, Wenzhou 325000, China; ^4^ Institute of Hepatology, Wenzhou Medical University, Wenzhou 325000, China; ^5^ Department of Infection Diseases, the First Hospital of Jiaxing, Jiaxing 314000, China

**Keywords:** hepatitis B virus reactivation, chemotherapy, nucleos(t)ide analogues prophylaxis, network meta-analysis, indirect comparison

## Abstract

**Objectives:**

Currently, no consensus exists regarding the optimal oral prophylactic regimens for hepatitis B surface antigen seropositive patients undergoing chemotherapy. We aimed to compare the efficacy of oral nucleos(t)ide analogues (NAs), including lamivudine, entecavir, adefovir, telbivudine and tenofovir, for the prevention of chemotherapy-induced hepatitis B virus (HBV) reactivation and its related morbidity and mortality in patients with chronic HBV (CHB) infection.

**Results:**

Fifty-two eligible articles consisting of 3892 participants were included. For HBV reactivation, prophylactic treatment with NAs were all significantly superior to no prophylaxis, with odds ratio (OR) from 0.00 (95% confidence interval [CI] 0.00~0.04) for the most effective intervention (tenofovir) to 0.10 (95% CI 0.06~0.14) for the least effective intervention (lamivudine). For secondary outcomes, prophylaxis with NAs also significantly outperformed observation. The results suggested that entecavir reduced the risk of HBV related hepatitis (predicted probability, 83%), HBV related death (68%) and all causes of hepatitis (97%) most efficaciously. It ranked second in decreasing all causes of death (34%).

**Materials and Methods:**

PubMed, Embase and Cochrane Library database were searched for controlled trials up to March 31, 2015. Primary outcome was the incidence of HBV reactivation. Secondary outcomes included the incidence of HBV-related hepatitis and death, all causes of hepatitis and death. Network meta-analysis combined direct and indirect evidence to estimate ORs for the clinical outcomes. A mean ranking and the probability of optimal therapeutic regime was obtained for each treatment based on clinical outcomes.

**Conclusions:**

Available evidence suggests that prophylatic therapy with tenofovir and entecavir may be the most potent interventions in prevention of HBV reactivation and HBV-related morbidity and mortality for CHB infection patients undergoing chemotherapy.

## INTRODUCTION

Hepatitis B virus (HBV) infection is a public and medical issue all over the world. Approximately 240 million people show serological evidence of chronic infection (hepatitis surface antigen [HBsAg] positive) [1]. Patients with chronic HBV (CHB) infection receiving immunosuppression treatment, such as oral corticosteroids, chemotherapy, immunosuppressors or hematopoietic stem cell transplantation (HSCT), may experience the risk of HBV reactivation, severe hepatitis and even life-threatening hepatic failure due to the suppression of immune system and enhancement of virus replication. Previous studies reported that the incidences of chemotherapy-induced HBV reactivation and HBV-related death in HBsAg positive patients were 19.32–85% and 2.27–33.33% [2–5], respectively. The serious complications of HBV reactivation in cancer patients can also cause disruption of chemotherapy, which may have negative effect on patients’ survival [6].

Because of the high rate of HBV reactivation-related morbidity and mortality in such individuals, there has been an increase in the awareness of the importance of prophylactic anti-HBV treatment during chemotherapy. Lamivudine, an oral nucleos(t)ide analog (NA), has been widely used for treating CHB infection in the last ten years. Lamivudine may inhibit HBV replication, decrease viral load in serum and improve hepatitis both clinically and histologically. A growing number of randomized controlled trials (RCTs) or retrospective studies have demonstrated that lamivudine prophylaxis can also improve the clinical outcome of HBsAg positive cancer patients undergoing chemotherapy with few adverse effects [2, 6–8]. According to APASL 2012 guidelines for management of CHB infection, lamivudine is the first-line pophylactic anti-HBV agent recommended for HBsAg positive patients at the initiation of cancer chemotherapy and its use is recommended to continue for at least 6 months after the completion of chemotherapy [9].

Currently, available NAs for clinical intervention include entecavir, adefovir, telbivudine and tenofovir in addition to lamivudine. All of these drugs can clear HBV or inhibit viral replication and improve clinical survival. Recently, several clinical studies have compared these different anti-HBV drugs for the prevention of HBV reactivation and its complications in CHB patients receiving chemotherapy [3, 10, 11]. Most trials used pairwise comparisons and only evaluated the prophylactic effect of one agent against lamivudine. Opinions differ concerning which oral NA is the most efficacious for prevention of HBV reactivation in CHB infection patients receiving chemotherapy.

Theoretically, RCTs with a large number of patient samples and multiple comparator arms should be conducted to answer this issue. However, this appears to be infeasible. To our knowledge, there has been no study systematically evaluating and comparing the prophylactic effects of these five anti-viral agents up to now because of a lack of evidence from head-to-head clinical trials. Network meta-analysis, which is also known as mixed-treatment comparison, may be a latent approach to solve the above problem. Compared with traditional meta-analysis, data from both direct and indirect comparisons can be synthesized by using network meta-analysis, which allows us to jointly compare several different therapeutic regimens. In view of the limitations of previous studies, we aimed to perform a systematic review and network meta-analysis to simultaneously compare the preventive effect of five oral NAs (entecavir, adefovir, telbivudine, tenofovir and lamivudine) for the prophylactic treatment of HBV reactivation and HBV- related morbidity and mortality in HBsAg positive patients undergoing chemotherapy.

## RESULTS

### Study characteristics

**Figure 1:**
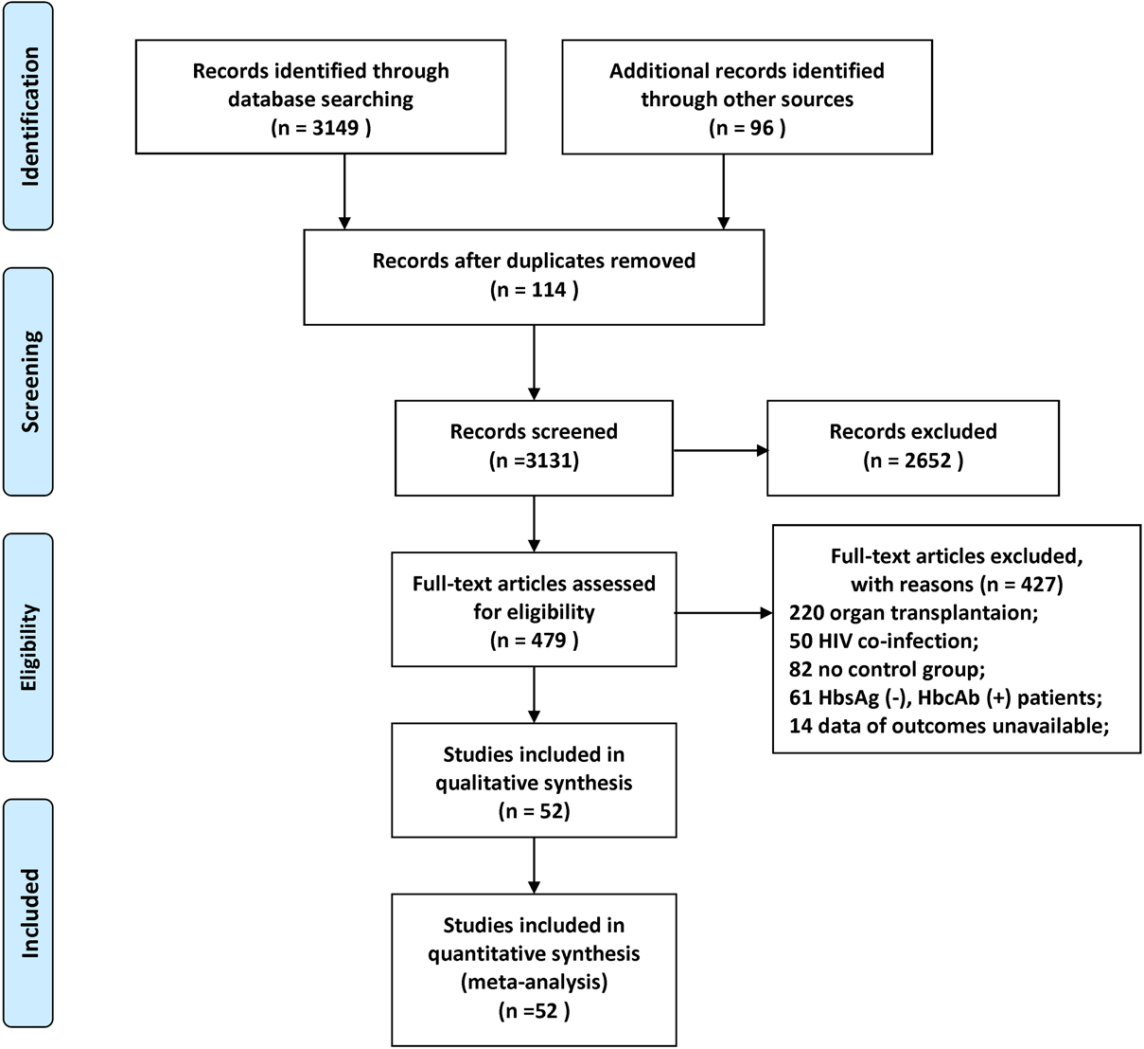
Literature search and selection

**Figure 2:**
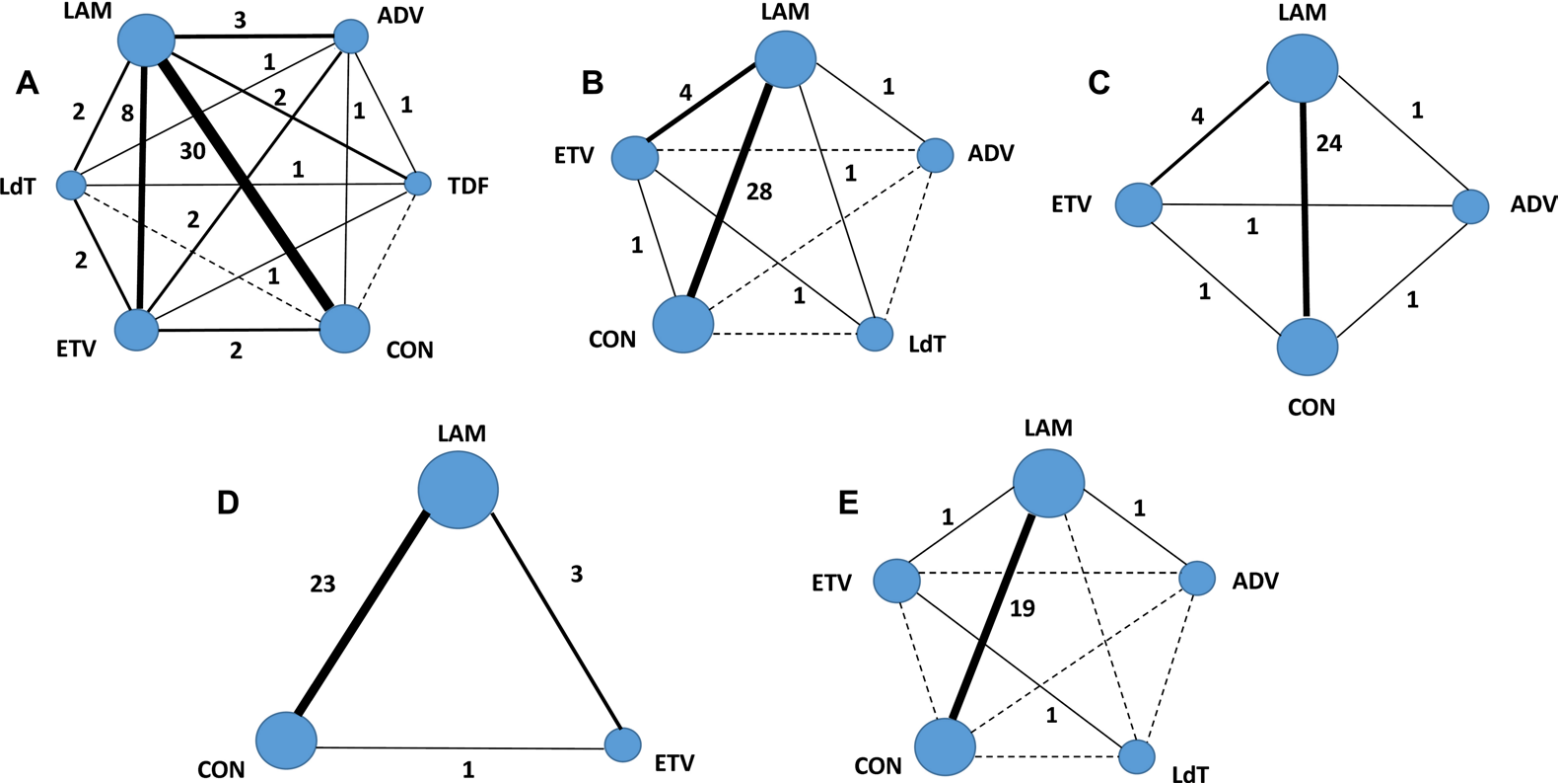
Evidence network of eligible comparisons for network meta-analysis The numbers along the link lines demonstrate the number of trials or pairs of trial arms. Each solid line represents direct comparisons between regimens that have been studied in head-to head (direct) comparisons in the eligible controlled trials. Each dashed line indicates that there is no direct comparison (indirect comparison) between two regimens. The width of the lines reflects the cumulative number of trials for each comparison and the size of every cycle is proportional to the number of included regimens (sample size). Different cycles represent different regimens accordingly. (**A**) HBV reactivation; (**B**) HBV-related hepatitis; (**C**) HBV-related death; (**D**) All causes of hepatitis; (**E**) All causes of death. CON = control (no prophylaxis); LAM = lamivudine; ETV = entecavir; ADV = adefovir; LdT = telbivudine; TDF = tenofovir.

Through the literature search and selection based on the criteria above, 479 articles were identified by reviewing 3245 potentially relevant publications. Then 427 publications were excluded after further assessment of the full text or abstracts. Finally 52 [2–8, 10–54] articles (53 trials) with a total of 3892 patients were deemed as suitable for the meta-analysis (Figure 1). All studies were two arm trials except for three trials with multiple comparator arms. Of these included trials, there were 6 RCTs; 35 retrospective cohort trials, 12 prospective cohort trials. The patients had various cancers: both solid tumors and hematological malignancies. The patients in six studies received allogeneic HSCT (allo-HSCT). As far as study sample size was concerned, the population size involved in the studies ranged from 11 to 258. A total of 2267 HBsAg positive patients were assigned to receive one of the five oral NAs as prophylaxis during chemotherapy or HSCT and 1625 HBsAg positive patients didn't receive prophylactic treatment. The prophylaxis initiated 0–7 day prior to chemotherapy and withdrawn 1–12 months after completion of chemotherapy. Detailed characteristics of the eligible studies were outlined in Table 1. The quality assessment and scores of prospective or retrospective cohort studies were summarized in Supplementary Table 1, which indicated that the quality of included studies was reliable. The results of quality assessment of RCTs suggested low to moderate risk of bias, which were summarized in Supplementary Figure 1. The geometric distribution of controlled trials on the primary and secondary outcomes were illustrated in Figure 2. Overall low heterogeneity and no significant publication bias were found among those pairwise comparisons of different prophylactic regimens (see Table 2 and Supplementary Figure 2).

**Table 1:** Characteristics of included studies

Study, Year [Reference]	Country	Types of cancer	intervention A vs. B	Treatment duration Median (range) or Mean ± SD	Intervention A vs. B			
					Age (y) Median (range) or Mean ± SD	Total No. (*n*)	Male No. (*n*)	Follow up (m) Median (range) or Mean ± SD
**Two-arms trials**								
**Randomized controlled trials**								
Lau et al., 2003 [6]	China HongKong	Lymphoma	LAM 100 mg/d vs. CON	Start: 7 days prior to chemo End: 6 weeks after the completion of chemo	50.6 (23~98) vs. 51.2 (24~98)	15 vs. 15	8 vs. 9	> 3
Jang et al., 2006 [8]	Korea	Hepatocellular carcinoma	LAM 100 mg/d vs. CON	Start: initiation of chemo End: 12 months after the completion of chemo	52.5 ± 8.4 vs. 53.2 ± 9.0	36 vs. 37	30 vs. 31	> 12
Hsu et al., 2008 [12]	China Taiwan	Non-Hodgkin's lymphoma	LAM 100 mg/d vs. CON	Start: day 1 of chemo End: 2 months after the completion of chemo	50.5(32–67) vs. 41 (20–74)	26 vs. 25	12 vs. 13	33.2 vs. 38.6 (median)
Long et al., 2011 [13]	China	Breast cancer	LAM 100 mg/d vs. CON	Start: 7 days prior to chemo End: 12 months after the completion of chemo	43 (20–62) vs. 45 (29–64)	21 vs. 21	0 vs. 0	N/A
Huang et al., 2014 [10]	China	Diffuse large B-cell lymphoma	LAM 100 mg/d vs. ETV 0.5 mg/d	Start: 7 days prior to chemo End: 6 months after the completion of chemo	44.5(25–76) vs. 41 (19–66)	60 vs. 61	37 vs. 31	40.7 (8.6–62.3)
Ho et al., 2015 [14]	China HongKong	Hematological/solid malignancies	ADV 10 mg/d vs. LAM 100 mg/d	Start: 7 days prior to chemo End: 6 months after the completion of chemo	51 (29–76) vs. 57 (21–82)	35 vs. 35	21 vs. 22	> 6
**Retrospective cohort study**								
Lim et al., 2002 [15]	Singapore	Hematological/solid malignancies	LAM 100 mg/d-300 mg/d vs. CON	Start: 5 days prior to chemo End: completion of chemo	47.5(25–75) vs. 54 (28–75)	16 vs. 19	12 vs. 10	11.5 (1–41) vs. 12 (0.5–49)
Persico et al., 2002 [16]	Italy	Non-Hodgkin's lymphoma	LAM 100 mg/d vs. CON	Start: during chemo End: 2 months after the completion of chemo	total 45 (38–61)	3 vs. 18	total 11	18
Lee et al., 2003 [4]	Korea	Non-Hodgkin's lymphoma	LAM 100 mg/d vs. CON	N/A	44 (29-68) vs. 47.5(18–70)	11 vs. 20	6 vs. 13	NA
Leaw et al., 2004 [17]	China Taiwan	aggressive lymphoma	LAM 100 mg/d vs. CON	Start: initiation of chemo End: 1 months after the completion of chemo	N/A	11 vs. 53	N/A	24 (2–120)
Nagamatsu et al., 2004 [5]	Japan	Hepatocellular carcinoma	LAM 100 mg/d vs. CON	Start: 28 days prior to chemo and continued throughout chemo	44 (29–68) vs. 46 (41–69)	8 vs. 9	6 vs. 7	N/A
Jia et al., 2004 [18]	China	Hematological/solid malignancies	LAM 100 mg/d vs. CON	Start: before chemo	N/A	8 vs. 8	Total 9	N/A
Ozguroglu et al., 2004 [19]	Turkey	Non-Hodgkin's lymphoma	LAM 100 mg/d vs. CON	Start: before chemo	44 (35–49) vs. 42.5 (14–72)	4 vs. 8	3 vs. 3	N/A
Lim et al., 2007 [20]	Singapore	Non-Hodgkin's lymphoma	LAM 100 mg/d vs. CON	End: 3-6 months after the completion of chemo	N/A	24 vs. 21	N/A	N/A
Chen et al., 2008 [21]	China	Allo-HSCT patients	LAM vs. CON	N/A	N/A	13 vs. 11	N/A	28.2(mean)
Tsutsumi et al., 2009 [22]	Japan	Non-Hodgkin's lymphoma	LAM vs. CON	N/A	N/A	10 vs. 15	N/A	N/A
Eren et al., 2009 [23]	Turkey	Hematological/solid malignancies	LAM 100 mg/d vs. CON	Start: not later than the first day of chemo End: 6 months after the completion of chemo	N/A	40 vs. 19	Total 35	N/A
Yeo et al., 2009 [24]	China Hongkong	Diffuse large B-cell lymphoma	LAM vs. CON	N/A	N/A	15 vs. 9	N/A	N/A
Koo et al., 2010 [25]	Singapore	Non-Hodgkin's lymphoma	LAM vs. CON	N/A	N/A	18 vs.8	N/A	N/A
Topcuogluet al., 2010 [26]	Turkey	Allo-HSCT patients	LAM 100mg/d vs. CON	Start: initiated with conditioning regimen End: 6-12 month after the cessation of immunosuppresion at posttransplant period	Total median 33	14 vs. 9	Total 22	N/A
Pei et al., 2010 [27]	China Taiwan	Non-Hodgkin's lymphoma	LAM vs. CON	Start: prior to chemo End: 0-9 month (range) months after cessation of rituximab treatment. (median 2month)	49 (31–72) vs. 54 (40–81)	5 vs.10	2 vs. 5	N/A
Sohn et al., 2011 [28]	Korea	Breast cancer	LAM vs. CON	before or during chemo prior to development of an apparent clinical hepatitis flare-up	48 (29–66) vs. 46 (23–75)	41 vs. 128	0 vs. 0	N/A
Yun et al., 2011 [29]	Korea	Breast cancer	LAM 100 mg/d vs. CON	Start: within 7 days prior to chemo	48 (30–68) vs. 46 (30–69)	55 vs. 76	0 vs. 0	N/A
Yan et al., 2012 [30]	China	Lung cancer	LAM vs. CON	Start: initiation of chemo	N/A	33 vs. 43	27 vs. 34	N/A
Mya et al., 2012 [31]	Singapore	multiple myeloma	LAM 100 mg/d vs. CON	Start: before chemo End: 6-12 months after the completion of chemo	N/A	11 vs. 4	N/A	median 33.6
Chen et al., 2012 [32]	China	Diffuse large B-cell lymphoma	LAM 100 mg/d vs. CON	Start: 7 days prior to chemo End: 3 months after the completion of chemo	47 (21–76) vs. 46.9(22–76)	30 vs. 20	19 vs. 11	N/A
Wang et al., 2013 [33]	China	Hematological/solid malignancies	LAM vs. CON	N/A	N/A	47 vs. 113	N/A	N/A
Lin et al., 2014 [2]	China	Lung cancer	LAM 100 mg/d vs. CON	Start: 7 days prior to chemo End: 3 months after completion of chemo	61.5(34–77) vs. 59 (30–79)	82 vs. 176	49 vs. 107	N/A
Lee et al., 2014 [7]	Korea	Breast cancer	LAM 100mg/d vs. CON	End: 0–28.9 month after the completion of chemo; median duration 7.5m (2.1–34.7m)	46 (29–67) vs. 45 (29–72)	73 vs.92	0 vs.0	49.7 (16.1–121.3) vs. 74 (23.5–140.6)
Nishida et al., 2013 [34]	Japan	Hematological/solid malignancies	ETV vs. CON	N/A	60 (43–79) vs. 59 (36–74)	8 vs. 29	2 vs. 10	25 (2–32) vs. 19 (4–102)
Li et al., 2011 [36]	China	Lymphoma	LAM 100 mg/d vs. ETV 0.5 mg/d	Start: 7 days prior to chemo	46 (20–81) VS. 44 (17–74)	89 vs. 34	52 vs. 22	N/A
Min et al., 2012 [37]	Korea	non-hepatic cancer	LAM vs. ETV	11.1 ± 8.2 vs. 11.5 ± 6.9	51.5 ± 9.4 vs. 48.2 ± 9.4	146 vs. 40	N/A	N/A
Chen et al., 2013 [35]	Australia	Haematological malignancies	LAM 100 mg/d vs. ETV 0.5 mg/d	N/A	N/A	11 vs. 4	N/A	36
Ling et al., 2013 [38]	Singapore	Solid malignancies	LAM vs. ETV	N/A	N/A	24 vs. 4	N/A	N/A
**Retrospective cohort study with historical control group**								
Lau et al., 2003 [39]	China Hongkong	Allo-HSCT patients	LAM 100 mg/d vs. CON		38.5(13–54) vs. 32 (5–48)	20 vs. 20	10 vs. 16	> 12
Li et al., 2006 [40]	China	Lymphoma	LAM 100 mg/d vs. CON	Start: 7 days prior to chemo End: 8 weeks after the completion of chemo	40 (16–74) vs. 41 (12–75)	40 vs. 116	26 vs. 72	> 3
Hsiao et al., 2006 [41]	China Taiwan	Allo-HSCT patients	LAM 100 mg/d vs. CON	Start: 0-62 weeks (range) prior to allo-HSCT, 11 weeks (median) End: posttransplantation period	41 (19–56) (LAM)	16 vs. 55	12 (LAM)	39 (2–216)
Cil et al., 2008 [42]	Turkey	Hematological/solid malignancies	LAM 100 mg/d vs. CON	Start: 7 days prior to chemo End: 2 months after the completion of chemo	44 (22–66) vs. 46(24–70)	37 vs. 50	23 vs. 32	31 (LAM)
Huang et al., 2009 [43]	China	Allo-HSCT patients	LAM 100 mg/d vs. CON	Start: 7 days prior to chemo End: 6 months after the completion of allo-HSCT	37 ± 12 vs. 29 ± 9	20 vs. 12	13 vs 7	12.3 vs. 43.8 (median)
**Prospective cohort studies**								
Shibole et al., 2002 [44]	Isreal	Lymphoma	LAM 150 mg/d vs. CON	Start: prior to initiation of chemo End: 6 months after the completion of chemo	55 (38–65) vs. 57.5(46–67)	7 vs. 4	4 vs. 4	N/A
Idilman et al., 2004 [45]	Turkey	Hematological/solid malignancies	LAM 100 mg/d vs. CON	Start: initiation of chemo End: 12 months after the completion of chemo	42 (35–68) vs. 40 (25–51)	8 vs. 10	5 vs. 4	17.1 (8–29) vs. 32.1 (5–59)
Tsai et al., 2011 [46]	China Taiwan	breast cancer	LAM 100 mg/d vs. CON	Start: 7 days prior to chemo End: 1 months after the completion of chemo	46.7 ± 9.2 vs. 50.4 ± 7.7	23 vs. 22	0 vs. 0	> 3
Kim et al., 2013 [3]	Asian	B-cell lymphoma	LAM vs. ETV	N/A	N/A	28 vs. 16	/	N/A
Chen et al., 2013 [11]	China Taiwan	Hematological/solid malignancies	LdT vs. ETV	Start: 7 days prior to chemo	53.9 ± 13.2 vs. 57.3 ± 13.9	48 vs. 24	22 vs. 15	mean 10.7 (0.57–27.8)
Gentile et al., 2014 [47]	Italy	Haematological malignancies	LAM vs. TDF	32 (9-72) vs. 24 (4-48)	N/A	13 vs. 25	N/A	N/A
**Prospective cohort studies with historical control group**								
Yeo et al., 2004 [48]	China Hongkong	Hematological/solid malignancies	LAM 100 mg/d vs. CON	Start: 7 days prior to chemo End: 2 months after completion of chemo	49 (35–77) vs. 49 (20–78)	65 vs. 193	34 vs. 82	2 months after the completion of chemo
Yeo et al., 2004 [49]	China Hongkong	Breast cancer	LAM 100 mg/d vs. CON	Start: 7 days prior to chemo End: 2 months after completion of chemo	46 (31–68) vs. 46 (31–71)	31 vs. 61	0 vs. 0	2 months after the completion of chemo
Dai et al., 2004 [50]	China Taiwan	Breast cancer	LAM 100 mg/d vs. CON	Start: 7 days prior to chemo End: 1 months after completion of chemo	47 (36–58) vs. 43 (27–55)	11 vs. 9	0 vs. 0	19 (11–25) vs. 10 (3–18)
Yeo et al., 2005 [51]	China Hongkong	Nasopharyngeal Carcinoma	LAM 100 mg/d vs. CON	Start: 7 days prior to chemo End: 2 months after completion of chemo	46.5(30–58) vs. 46 (40–65)	16 vs. 21	14 vs. 15	2 months after the completion of chemo
Hui et al., 2005 [52]	China Hongkong	Allo-HSCT patients	LAM vs. CON	Start: 7 days prior to allo-HSCT End: 52 weeks after allo-HSCT or until death	42 (23–38) (LAM)	19 vs. 14	10(LAM)	N/A
**Multi-arms trials**								
**Retrospective cohort study**								
Kim et al., 2013 [3]	Asian	B-cell lymphoma	ADV vs. ETV vs. LAM vs. CON	N/A	N/A	7 vs. 31 vs. 96 vs. 22	N/A	N/A
Choi et al., 2014 [53]	Korea	Hematological/solid malignancies	LAM vs. ETV vs. ADV vs. LDT vs. TDF	N/A	N/A	77 vs. 87 vs. 17 vs. 14 vs. 9	N/A	median 16.4 months following the start of chemo
**Prospective cohort study**								
Yoo et al., 2012 [54]	Korea	Hematological/solid malignancies	LAM vs. ETV vs. LDT	N/A	N/A	86 vs. 31 vs. 124	Total 129	> 6

CON = control (no prophylaxis); LAM = lamivudine; ETV = entecavir; ADV = adefovir; LdT = telbivudine; TDF = tenofovir; Allo-HSCT = allogeneic hematopoietic stem cell transplantation.

**Table 2:** Assessment of heterogeneity for direct comparisons and comparison of outcomes between pair-wise meta-analysis and network meta-analysis

Treatment Comparisons	Results of Pair-Wise Meta-Analysis	*I*^2^%	Results of Network Meta-Analysis
**HBV reactivation**			
ETV vs. ADV	0.38 (0.02, 6.10)	56.3	0.29 (0.09, 1.06)
LAM vs. ADV	1.36 (0.61, 3.03)	0	1.30 (0.49, 3.81)
LAM vs. ETV	3.67 (1.72, 7.82)	35.2	4.38 (2.06, 9.53)
ETV vs. CON	0.19 (0.00, 18.73)	86.8	0.02 (0.01, 0.05)
LdT vs. ETV	1.60 (0.17, 15.42)	100	1.89 (0.46, 8.50)
TDF vs. ETV	0.98 (0.05, 19.57)	N/A	0.17 (0.00, 2.03)
CON vs. LAM	7.47 (5.30, 10.52)	0	10.47 (7.03, 16.55)
LdT vs. LAM	0.44 (0.11, 1.83)	40.5	0.43 (0.12, 1.74)
TDF vs. LAM	0.07 (0.01, 0.66)	N/A	0.04 (0.00, 0.43)
**HBV-related hepatitis**			
ADV vs. LAM	2.06 (0.18, 23.83)	N/A	2.21 (0.17, 82.04)
LAM vs. ETV	4.09 (1.13, 14.77)	8.4	5.36 (1.78, 19.97)
CON vs. ETV	0.48 (0.04, 6.11)	N/A	46.71 (14.33, 185.10)
LdT vs. ETV	4.60 (0.26, 81.82)	N/A	2.43 (0.48, 14.64)
CON vs. LAM	6.89 (4.83, 9.83)	0	8.59 (5.84, 13.35)
LdT vs. LAM	0.39 (0.15, 0.98)	N/A	0.43 (0.12, 1.88)
**HBV- related death**			
LAM vs. ADV	0.73 (0.04, 14.88)	N/A	1.26 (0.15, 34.78)
CON vs. ADV	1.76 (0.17, 18.32)	N/A	3.60 (0.42, 96.28)
ETV vs. LAM	1.32 (0.12, 15.03)	N/A	0.32 (0.04, 1.65)
CON vs. ETV	8.22 (0.95, 81.99)	N/A	8.88 (1.73, 90.38)
CON vs. LAM	2.51(1.54, 4.08)	0	2.84 (1.66, 4.86)
**All causes of hepatitis**			
LAM vs. ETV	3.44 (1.51, 7.82)	0	2.81 (0.92, 8.09)
CON vs. ETV	0.48 (0.04, 6.11)	N/A	12.12 (4.00, 39.36)
CON vs. LAM	3.96 (2.79, 5.63)	29.9	4.34 (3.01, 6.73)
**All causes of death**			
LAM vs. ADV	1.00 (0.36, 2.74)	N/A	1.01 (0.18, 5.71)
LAM vs. ETV	0.76 (0.07, 8.65)	N/A	0.95 (0.06, 35.24)
ETV vs. LdT	1.68 (0.57, 4.97)	N/A	1.72 (0.28, 10.02)
CON vs. LAM	2.35 (1.51, 3.66)	11.8	2.83 (1.68, 5.01)

CON = control (no prophylaxis); LAM = lamivudine; ETV = entecavir; ADV = adefovir; LdT = telbivudine; TDF = tenofovir.

### HBV reactivation

Fifty-six comparisons assessed the efficacy of the whole 6 interventions to reduce the incidence of HBV reactivation. Overall, 1174 patients (42.43%) were assigned to lamivudine prophylaxis, 276 (9.97%) to entecavir prophylaxis, 59 (2.13%) to adefovir prophylaxis, 138 (4.99%) to telbivudine prophylaxis, 34 (1.23%) to tenofovir prophylaxis and 1086 (39.25%) patients did not receive any prophylactic intervention.

**Figure 3:**
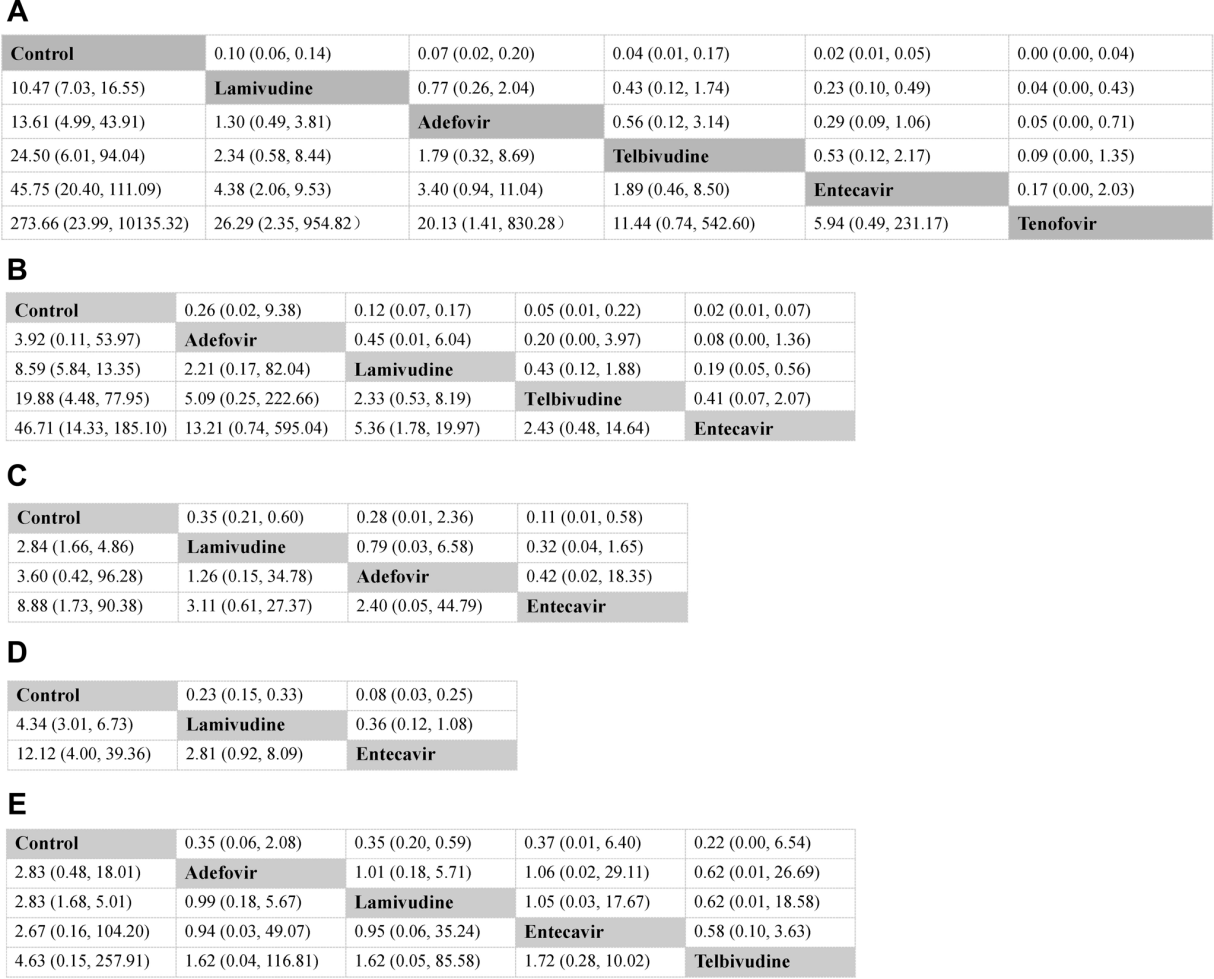
Major clinical efficacy of all interventions according to network meta-analysis Treatments are reported in efficacy order, which is from the least to the most efficacious treatment. The odds ratios (ORs) were estimated in upper and lower triangle comparing column-defining with row-defining treatment. ORs lower than 1 favors the column-defining treatment. Numbers in parentheses indicate 95% confidence intervals. (**A**) HBV reactivation; (**B**) HBV-related hepatitis; (**C**) HBV-related death; (**D**) All causes of hepatitis; (**E**) All causes of death.

**Figure 4:**
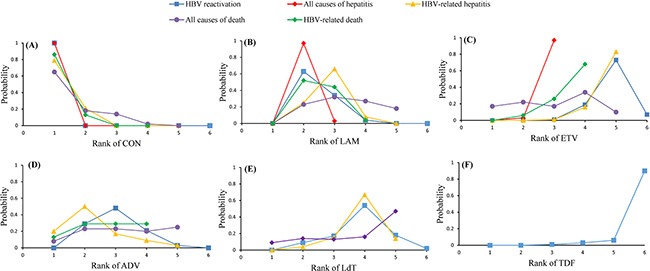
Rankograms showing probability of each strategy having each specific rank (1–6) for HBV reactivation, HBV-related hepatitis, HBV-related death, all causes of hepatitis and all causes of death Ranking indicates the probability to be the best treatment, the second best, the third best and so on. Rank 1 is worst and rank N is best. (**A**) Rank of CON; (**B**) Rank of LAM; (**C**) Rank of ETV; (**D**) Rank of ADV; (**E**) Rank of LdT; (**F**) Rank of TDF. CON = control (no prophylaxis); LAM = lamivudine; ETV = entecavir; ADV = adefovir; LdT = telbivudine; TDF = tenofovir.

Figure 3A illustrated the odds ratios (ORs) with 95% confidence interval (CI) of outcomes obtained from the network meta-analysis. All active interventions demonstrated significant superiority over no prophylactic therapy for reducing the incidence of HBV reactivation. In the comparisons between different active interventions, both entecavir (OR 0.23, 95% CI 0.10 ~ 0.49) and tenofovir (OR 0.04, 95% CI 0.00 ~ 0.43) were significantly better than lamivudine. Tenofovir significantly provided a more favorable outcome than adefovir (OR 0.05, 95% CI 0.00 ~ 0.71). Although statistical significance was not reached for other comparisons, there was a trend that entecavir was superior to adefovir (OR 0.29, 95% CI 0.09 ~ 1.06) and telbivudine (OR 0.53, 95% CI 0.12 ~ 2.17), while tenofovir was superior to entecavir (OR 0.17, 95% CI 0.00 ~ 2.03) and telbivudine (OR 0.09, 95% CI 0.00 ~ 1.35). Figure 4A–4F showed the distribution of probabilities of each prophylactic interventions being ranked at each of the possible six positions. Tenofovir had the highest probabilities (90.0%) for HBV reactivation rate reduction followed by entecavir with the second highest probability (73.0%).

### HBV-related hepatitis

A total of 36 comparisons, including 5 interventions were available to this analysis. Overall, 1164 patients (42.91%) were assigned to lamivudine prophylaxis, 178 (6.56%) to entecavir prophylaxis, 35 (1.29%) to adefovir prophylaxis, 124 (4.57%) to telbivudine prophylaxis, and 1212 (44.67%) patients did not receive any prophylactic intervention.

Figure 3B showed the result of the direct and indirect comparisons of the included regimens for this outcome. Significant efficacy for all active interventions could be observed when compared with control, with the exception of adefovir. In the comparisons between four anti-HBV interventions, entecavir achieved a significantly positive clinical outcome when compared with lamivudine (OR 0.19, 95% CI 0.05 ~ 0.56). In addition, although not differing significantly, entecavir also tended to show greater beneficial effects than adefovir (OR 0.08, 95% CI 0.00 ~ 1.36) or telbivudine (OR 0.41, 95% CI 0.07 ~ 2.07). Figure 4A–4E showed the distribution of probabilities of each prophylactic interventions being ranked at each of the possible five positions. Entecavir had the highest probabilities (83%) of with respect to reducing the incidence of HBV-related hepatitis.

### HBV-related death

Thirty-two comparisons, including 4 interventions contributed to this analysis. A total of 729 patients (43.19%) were assigned to lamivudine prophylaxis, 85 (5.04%) to entecavir prophylaxis, 7 (0.41%) to adefovir prophylaxis and 867 (51.36%) patients did not receive any prophylactic treatment.

Figure 3C showed the pooled estimates for the outcome of reducing the incidence of HBV-related death in network meta-analysis. Both lamivudine (OR 0.35, 95% CI 0.21 ~ 0.60) and entecavir (OR 0.11, 95% CI 0.01 ~ 0.58) showed significant superiority over control. Although not differing significantly, adefovir also appeared to be more favorable than control (OR 0.28, 95% CI 0.01 ~ 2.36). When compared with each active intervention, none of the comparisons reached statistical significance. However, entecavir was more likely to induce a more favorable clinical outcome than lamivudine (OR 0.32, 95% CI 0.04 ~ 1.65) or adefovir (OR 0.42, 95% CI 0.02 ~ 18.35). Figure 4A–4D showed the distribution of probabilities of each prophylactic interventions being ranked at each of the possible four positions. Entecavir was ranked the most efficacious intervention with regard to reducing the incidence of death due to HBV reactivation.

### All causes of hepatitis

Twenty-seven comparisons, including 3 interventions contributed to analyze the outcome of all causes of hepatitis. A total of 918 patients (41.33%) were assigned to lamivudine prophylaxis, 147(6.62%) to entecavir prophylaxis and 1156 (52.05%) patients did not receive any prophylactic regimes.

Figure 3D showed the result of the network meta-analysis for this outcome. Patients receiving lamivudine (OR 0.23, 95% CI 0.15 ~ 0.33) or entecavir (OR 0.08, 95% CI 0.03 ~ 0.25) prophylactic treatment could significantly prevent the incidence of hepatitis when compared with no prophylactic intervention. Entecavir was associated with a superior outcome over lamivudine (OR 0.36, 95% CI 0.12 ~ 1.08) although this did not achieve statistical significance. Figure 4A–4C showed the distribution of probabilities of each prophylactic interventions being ranked at each of the possible three positions. On the whole, entecavir was demonstrated to be the most efficacious treatment among three interventions as entecavir had the highest probabilities (97%) in terms of hepatitis rate reduction as shown in Figure 4C.

### All causes of death

A total of 22 comparisons, including 5 interventions provided data for the analyses of this outcome. 548 patients (39.68%) were assigned to lamivudine prophylaxis, 58 (4.20%) to entecavir prophylaxis, 35 (2.53%) to adefovir prophylaxis, 48 (3.48%) to telbivudine prophylaxis and 692 (50.11%) patients did not receive any prophylactic intervention.

Figure 3E illustrated the pooled estimates for the outcome of reducing the incidence of all causes of death. Lamivudine prophylaxis was statistically better than no prophylaxis (OR 0.35, 95% CI 0.20 ~ 0.59). Other comparisons showed no statistical significance. However, there was a trend that all active interventions were better than control and telbivudine appeared to be more effective than lamivudine (OR 0.62, 95% CI 0. 01 ~ 18.58), adefovir (OR 0.62, 95% CI 0.01 ~ 26.69) as well as entecavir (OR 0.58, 95% CI 0.10 ~ 3.63). Figure 4A–4E showed the distribution of probabilities of each prophylactic interventions being ranked at each of the possible five positions. Telbivudine had the greatest probability (47%) for being the best prophylactic option on reducing the incidence of overall death. The treatment of entecavir (34%) shows the highest probability for being in the second ranking positions.

### Comparisons between traditional pairwise and Bayesian network meta-analyses

Table 2 showed the results of traditional pairwise and Bayesian network meta-analyses. Although the pooled estimates for the outcome showed small differences, the 95% CIs from traditional pairwise meta-analysis and network meta-analysis in general overlapped. Assessment of inconsistency by node splitting method between direct and indirect evidence was summarized in Table 3. The node-splitting method demonstrated inconsistency within the networks for the outcome of HBV-related hepatitis. No significant inconsistency within the networks for most treatment comparisons of the other three outcomes. As for the outcome of all causes of death, no closed loops were found. Therefore, inconsistency between direct and indirect evidence could not be assessed by the node splitting method, although the results of direct and indirect comparisons could be compatible for this outcome.

**Table 3:** Assessment of inconsistency between direct and indirect evidence

Treatment comparisons	*P* value of node-splitting method
**HBV reactivation**	
ADV vs. CON	0.27
ADV vs. ETV	0.61
LAM vs. CON	0.15
ETV vs. CON	0.4
ETV vs. TDF	0.07
ADV vs. TDF	0.11
ADV vs. LAM	0.77
ETV vs. LAM	0.22
**HBV-related hepatitis**	
ETV vs. LAM	0.01
ETV vs. CON	0.02
LAM vs. CON	0.02
**HBV-related death**	
ADV vs. ETV	0.02
ADV vs. LAM	0.05
ADV vs. CON	0.08
ETV vs. LAM	0.90
ETV vs. CON	0.77
LAM vs. CON	0.77
**All causes of hepatitis**	
ETV vs. LAM	0.08
ETV vs. CON	0.06
LAM vs. CON	0.09

CON = control (no prophylaxis); LAM = lamivudine; ETV = entecavir; ADV = adefovir; LdT = telbivudine; TDF = tenofovir.

## DISCUSSION

In this network meta-analysis, we evaluated the prophylactic efficacy of five oral NAs on chemotherapy-induced HBV reactivation and its related morbidity and mortality, including HBV-related hepatitis and death, all causes of hepatitis and mortality, in cancer patients with CHB infection. We found that tenofovir was the most effective agent to prevent HBV reactivation and prophylaxis with entecavir appeared to be the most potent intervention to reduce the incidence of HBV related morbidity and mortality.

It is well-known that liver injury resulting from HBV reactivation goes through two stages in HBV carrier receiving chemotherapy [55]. In the initial stage, chemotherapy-induced immune suppression may lead to remarkable HBV replication. Serum levels of HBV-DNA, HBV DNA polymerase, and hepatitis Be antigen (HBeAg) significantly increase and as a result, infected hepatocytes may suffer direct damage. The second stage comprises reconstitution of immune function after withdrawal of chemotherapy. The exaggerated immune response may mediate severe injury of infected hepatocytes, which clinically manifests as various HBV reactivation related complications, including hepatitis, liver failure and even death.

Since liver damage is closely relevant to HBV reactivation, prophylactic antiviral treatment during chemotherapy has been applied in the last decade. Lamivudine was initially available to prevent HBV reactivation clinically and several meta-analyses assessed the overall benefits of preventive lamivudine therapy in this population. Loomba et al. [56] showed that compared with no prophylaxis, lamivudine could significantly reduce the risk of HBV reactivation, HBV-associated hepatitis by 79% in HBsAg positive patients who underwent chemotherapy. Similarly, a traditional meta-analysis [57] including four RCTs also showed that lamivudine conferred a significantly more efficacious antiviral prophylaxis when compared with no prophylactic agent. With regards to other oral NAs, large scale RCTs with multiple comparator or meta-analysis have not been yet conducted to compare the effectiveness of different prophylactic interventions. For one RCT [10], prophylaxis with entecavir had a beneficial effect on lowering incidence with HBV-related hepatitis and HBV reactivation when compared with lamivudine among diffuse large B-cell lymphoma patients with CHB infection during chemotherapy. Another RCT [14] demonstrated that compared with lamivudine, the addition of adefovir resulted in similar efficacy in preventing chemotherapy-related HBV reactivation in CHB infected patients. The findings of these previous pairwise meta-analysis or RCTs are consistent with our findings.

Our meta-analysis has several strengths. Firstly, this is the largest meta-analysis with respect to this issue to date and we are confident that all correlative studies, including RCTs and cohort studies, have been properly assessed after extensive literature search and review. Furthermore, to our knowledge, this is also the first study which systematically assessed the prophylactic effect of all available oral NAs among CHB infection patients during chemotherapy. A traditional meta-analysis allows for only a direct comparison of individual pairs of intervention. However, this network meta-analysis combined total 3892 patients who received different prophylactic interventions and explored the effect of both direct and indirect comparisons between multiple interventions in a single analysis. In addition, after performing a Bayesian meta-analysis, we provided a rank order for prophylaxis strategies based on their capacity to reduce the incidence of HBV reactivation and HBV related events, which may provide an up to date scientific evidence to support clinicians in selecting oral NAs. Lastly, we conducted an inconsistency diagnostic analysis by node-splitting method for all loops to decrease concerns regarding potential inconsistency.

However, the results from our network meta-analysis need to be interpreted with caution for several reasons. First, there was some risk of bias in the included studies in terms of study design. Due to limited published RCTs, we also included the prospective and retrospective cohort studies for our study. But the methodological quality of these cohort studies was moderate to high by quality assessment. Besides that, heterogeneity of patients’ characteristics, such as patient populations, definition of clinical outcome, duration of prophylaxis and follow-up, time of HBV reactivation and chemotherapy regimens used, also might result in bias in the analysis. However, the results of heterogeneity tests suggested low heterogeneity, which we believe are acceptable. Secondly, because of the low number of head-to-head clinical trials comparing other anti-HBV agents except for lamivudine, the validity of our conclusions may be incomplete. Although tenofovir was suggested as the optimum treatment for primary outcome in this study, we were not able to assess the efficiency of tenofovir for secondary outcomes due to a lack of relevant data from the included trials. So we would recommend caution when interpreting the present conclusions. Thirdly, sample sizes assigned to each pair-wise comparison were small in many included studies. Fourthly, due to be unavailability of comprehensive data in most studies, we could not reliably evaluate the adverse effect of the different drugs.

In summary, this network meta-analysis suggests that chemotherapy with anti-HBV prophylaxis confers significant benefit over observation for patients with CHB infection. Among available NAs, tenofovir and entecavir may be the most effective treatment for prevention of chemotherapy-induced HBV reactivation and HBV-related morbidity and mortality.

## MATERIALS AND METHODS

### Literature search

We conducted a computerized literature search of PubMed, Embase, the Cochrane Library (prior to Mar 31, 2015) with the following keywords and subject terms: [“hepatitis B” (MeSH) or hepatitis B or HBV] and [reactivation] and [“lamivudine” (MeSH) or “entecavir“(MeSH) or “adefovir” (MeSH) or “telbivudine” (MeSH) or “tenofovir” (MeSH)]. The literature search strategy was in accordance with PRISMA (Preferred Reporting Items for Systematic Reviews and Meta-Analyses) guideline [58]. References of retrieved articles and meeting abstracts were also screened. Case reports, editorials, letters and review articles were excluded. In case that a publication overlapped with other publication of the same trial, only the article with more details or the most recent article was adopted.

### Selection criteria

Studies included in our meta-analysis satisfied all the following criteria: (1) studies were RCTs or retrospective or prospective cohort studies with controls (concurrent or historical); (2) study population was hematological or solid cancers patients with HBsAg positive undergoing chemotherapy or HSCT; (3) studies assessed the effectiveness of prophylactic therapy with one or more of the five oral NAs, including lamivudine, entecavir, adefovir, telbivudine and tenofovir, on prevention of HBV reactivation or HBV-related morbidity and mortality during chemotherapy. Exclusion criteria were: (1) non-cancer patients receiving immunosuppression therapy, including organ transplantation, inflammatory bowel disease and autoimmune diseases patients; (2) patients co-infecting human immunodeficiency virus (HIV) or other hepatitis viruses (hepatitis C virus [HCV], hepatitis D virus [HDV]); (3) patients with past HBV infection [HBsAg negative and hepatitis B core antibody (HBcAb) positive]; (4) patients receiving oral NAs treatment before. When a paper did not provide relevant data, or the case that the provided data were not sufficient, we contacted the corresponding authors by e-mail to obtain the raw data.

The primary outcome of this study was incidence of HBV reactivation, which was defined as an increase in HBV-DNA level to 10-fold or more when compared with baseline level, or appearance of HBV-DNA in previously negative patient or an absolute increase of HBV-DNA that exceeded 10^9^ ge/mL in the absence of other systemic infection [59]. The secondary outcome measures included: (1) all causes of hepatitis, defined as more than 3-fold increase in alanine aminotransferase (ALT) that exceeded the upper normal limit (UNL) or an absolute increase of ALT to over 100 IU/L; (2) HBV-related hepatitis, defined as more than 3-fold increase in ALT that exceeded UNL and a 10-fold increase in serum HBV- DNA level in the absence of other apparent causes of hepatitis; (3) all causes of death; (4) HBV-related death, defined as death of a patient who had documented HBV reactivation without other apparent causes of death.

### Data extraction and study quality

Two reviewers (Min-Yue Zhang, Gui-Qi Zhu) independently assessed the full manuscripts or abstracts of eligible studies and extracted data and outcomes using an electronic standard form. The following information from each study was summarized: (1) first author, (2) year of publication, (3) country, (4) study design, (5) types of cancers, (6) prophylactic interventions and (7) patients’ characteristics, including ages, total numbers of patients, numbers of male patients, numbers of events, duration of prophylaxis and follow up. Any conflicts regarding data extraction were resolved by an additional investigator, Ming-Hua Zheng.

### Quality assessment

Two reviewers (Min-Yue Zhang, Gui-Qi Zhu) independently assessed the quality of each included studies. Newcastle-Ottawa Quality Assessment Scale was adopted to assess the methodological quality of prospective or retrospective cohort studies. Three major components of each study, including patient selection, comparability of interventions and observation group, and assessment of outcome, were examined (Supplementary Table 2). Cochrane Risk of Bias Tool was adopted to assess the methodological quality of included RCTs [60]. This tool included the following items: sequence generation for the randomization of subjects, allocation of concealment of treatment, blinding, incomplete outcome data, selective outcome reporting and other sources of bias. Trials with high or unclear risk for bias for any one of the first three components were considered as trials with high risk of bias. Otherwise, they were regarded as trials with low risk of bias.

### Data analysis

Firstly the pairwise meta-analysis was conducted using Stata software (version 10.0, StataCorp, College Station, TX). Then a network meta-analysis within a Bayesian framework was conducted using Markov chain Monte Carlo methods in WinBUGS (Medical Research Council Biostatistics Unit, Cambridge, United Kingdom). The methods of pairwise meta-analysis and network meta-analysis were detailed in our previous publications [61–65].

## SUPPLEMENTARY MATERIALS FIGURES AND TABLES



## Abbreviations

NA: nucleos(t)ide analogue
HBV: hepatitis B virus
CHB infection: chronic HBV infection
OR: odds ratio
CI: confidence interval
HBsAg: hepatitis B surface antigen
HBeAg: hepatitis B eantigen
ALT: alanine aminotransferase
Allo-HSCT: allogeneic hematopoietic stem cell transplantation
RCT: randomized controlled trial
HIV: human immunodeficiency virus
HCV: hepatitis C virus
HDV: hepatitis D virus
HBcAb: hepatitis B core antibody
UNL: upper normal limit
CON: control
LAM: lamivudine
ETV: entecavir
ADV: adefovir
LdT: telbivudine
TDF: tenofovir.
