# Supplementary material for: Systematic review with network meta-analysis: Comparative efficacy of oral nucleos(t)ide analogues for the prevention of chemotherapy-induced hepatitis B virus reactivation
Source: Oncotarget. 2016 Apr 21;7(21):30642–58. doi: 10.18632/oncotarget.8907 (PMC5058707; doi:10.18632/oncotarget.8907)
Supplement: Supplementary file 1 [file oncotarget-07-30642-s001.pdf]

Systematic review with network meta-analysis: Comparative efficacy of oral nucleos(t)ide analogues for the prevention of chemotherapy-induced hepatitis B virus reactivation

Supplementary Materials

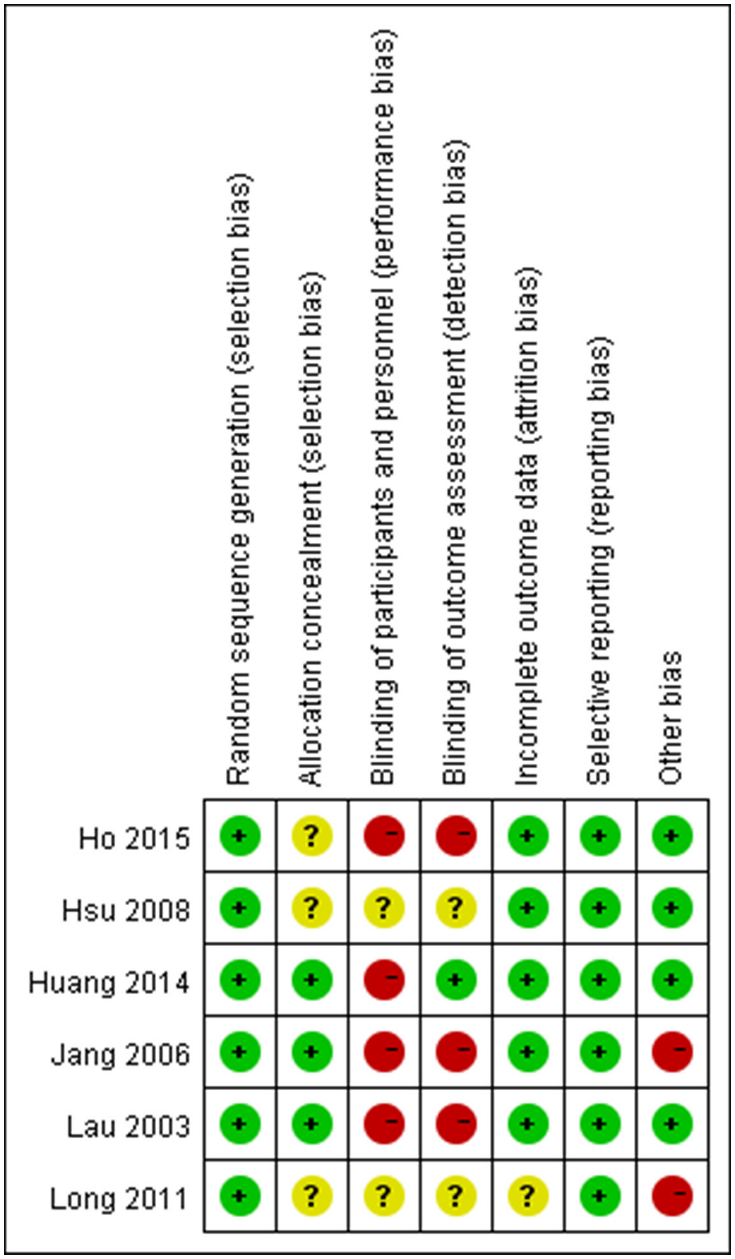

Supplementary Figure S1: Summary for Cochrane risk of bias tool results. The green symbols represent low risk of bias, the yellow symbols represent unclear risk of bias and the red symbols represent high risk of bias.

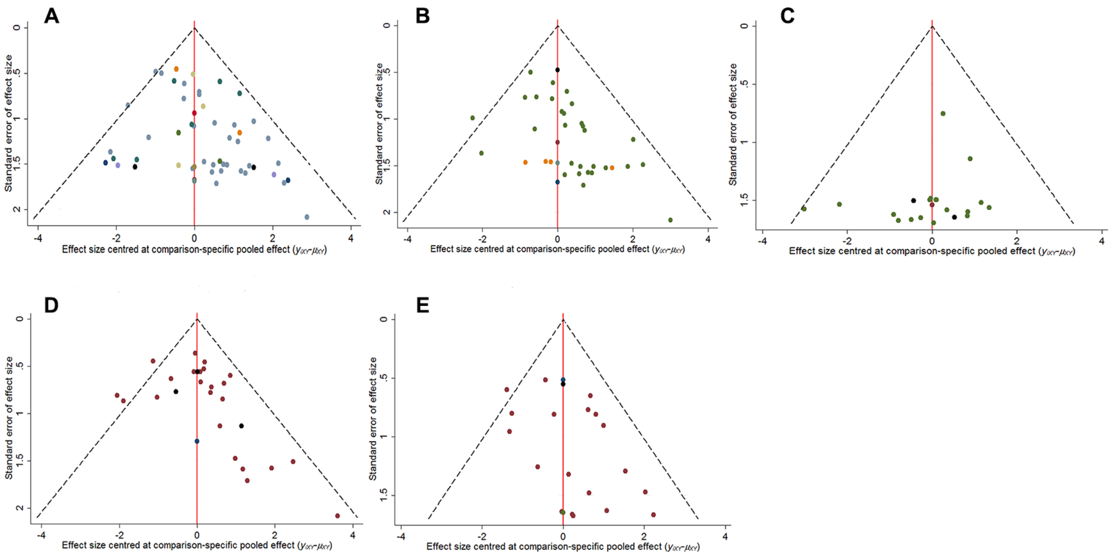

Supplementary Figure S2: Comparison-adjusted funnel plot for the prophylactic treatments network in terms of clinical outcomes. (**A**) HBV reactivation; (**B**) HBV-related hepatitis; (**C**) HBV-related death; (**D**) All causes of hepatitis; (**E**) All causes of death. The red line represents the null hypothesis that the study-specific effect sizes do not differ from the respective comparison-specific pooled effect estimates. Different colors correspond to different comparisons. Estimates below 1 indicate that the benefit of the experimental intervention is more pronounced in the trial than the pooled estimate. Observations from small studies missing on the right side of the line of null effect (ratio of rate ratios > 1) indicate that small studies tend to exaggerate the effectiveness of experimental treatments.

Supplementary Table S1: Quality assessment of included prospective or retrospective cohort studies

Authors [Reference]	Year	Selection			Comparability		Outcome Assessment		Score
		1	2	3	4	5	6	7	
Chen [11]	2013	*	*	*	*	*	*	*	*******
Chen [21]	2008	*	*	*	*	*	*		******
Chen [32]	2012	*	*	*	**	*	*		*******
Chen [35]	2013	*	*	*	**	*	*		*******
Choi [53]	2014	*	*	*	**	*	*		*******
Cil [42]	2008	*	*	*	**	*			******
Dai [50]	2004	*	*	*	**		*	*	*******
Eren [23]	2009	*	*	*	**	*	*		*******
Gentile [47]	2014	*	*	*	*	*	*		******
Ho [14]	2015	*	*	*	**	*	*		*******
Hsiao [41]	2006	*	*	*	**	*		*	*******
Hsu [12]	2008	*	*	*	**	*	*	*	********
Huang [43]	2009	*	*	*	**	*	*		*******
Huang [10]	2014	*	*	*	**	**	*		********
Hui [52]	2005	*	*	*	**	*	*		*******
Idilman [45]	2004	*	*	*	**	**	*		********
Jang [8]	2006	*	*	*	**	*	*		*******
Jia [18]	2004	*	*	*	**	*	*	*	********
Kim [3]	2013	*	*	*	**	*	*	*	********
Koo [25]	2010	*	*	*	**	**	*	*	*********
Lau [39]	2002	*	*	*	*	*			*****
Lau [6]	2003	*	*	*	**	*	*		*******
Leaw [17]	2004	*	*	*	**	*	*		*******
Lee [4]	2003	*	*	*	**	*	*		*******
Lee [7]	2014	*	*	*	**	*			******
Li [40]	2006	*	*	*	*	*	*		******
Li [36]	2011	*	*	*	**	*	*		*******
Lim [15]	2002	*	*	*	**	*	*	*	********
Lim [20]	2007	*	*	*	**	**			*******
Lin [2]	2014	*	*	*	*	*	*	*	*******

Ling [38]	2013	*	*	*	**	*	*	*	********
Long [13]	2011	*	*	*	**	*	*		*******
Min [37]	2012	*	*	*	*	*	*		******
Mya [31]	2012	*	*	*	**	**	*	*	********
Nagamatsu [5]	2004	*	*	*	**	**	*		********
Nishida [34]	2013	*	*	*	**	*	*	*	********
Ozguroglu [19]	2004	*	*	*	**	**	*		********
Pei [27]	2010	*	*	*	*	*	*	*	*******
Persico [16]	2002	*	*	*	**	**	*	*	*********
Shibolet [44]	2002	*	*	*	**	*	*		*******
Sohn [28]	2011	*	*	*	**	**		*	********
Topcuoglu [26]	2010	*	*	*	**	*	*		*******
Tsai [46]	2010	*	*	*	**	*	*	*	********
Tsutsumi [22]	2008	*	*	*	**	*	*		*******
Wang [33]	2013	*	*	*	**	*	*	*	********
Yan [30]	2012	*	*	*	**	*	*		*******
Yeo [48]	2004	*	*	*	*	**	*		*******
Yeo [49]	2004	*	*	*	**	*	*	*	********
Yeo [51]	2005	*	*	*	**	*	*		*******
Yeo [24]	2009	*	*	*	**	*		*	*******
Yoo [54]	2012	*	*	*	**	*	*		*******
Yun [29]	2011	*	*	*	**	*	*	*	********

Supplementary Table S2: Checklist for quality assessment and scoring of prospective or retrospective cohort studies

Check List

Selection
1. Assignment for treatment: any criteria reported? (if yes, one star)
2. How representative was the prophylaxis group in comparison for the prevention of chemotherapy-inducing hepatitis B virus reactivation? (if yes, one star; no star if the patients were selected or selection of group was not described)
3. How representative was the observation group in comparison for the prevention of chemotherapy-inducing hepatitis B virus reactivation? (if drawn from the same community as the treatment group, one star; no star if drawn from a different source or selection of group was not described)
Comparability
4. Group comparable for 1, 2, 3, 4 (if yes, two stars; one star was assigned if one of these four characteristics was not reported even if there were no other differences between the two groups and other characteristics had been controlled for; no star was assigned if the two groups differed)
5. Group comparable for 5, 6, 7 (if yes, two stars; one star was assigned if one of these two characteristics was not reported even if there were no other differences between the two groups and other characteristics had been controlled for; no star was assigned if the two groups differed)
Outcome assessment
6. Clearly defined outcome of interest (yes, one star for information ascertained by record or interview; no star if this information was not reported)
7. Adequacy of follow-up (one star if follow-up > 90%)

Comparability variables: 1 = patients with cancer; 2 = patients with HBsAg positive; 3 = type of cancer or basic disease; 4 = chemotherapy or HSCT for prophylaxis; 5 = primary outcomes; 6 = patients' hepatitis viral marker; 7 = secondary outcomes.
